# Why Behavioral Indicators May Fail to Reveal Mental States: Individual Differences in Arousal-Movement Pattern Relationships

**DOI:** 10.3389/fpsyg.2019.00270

**Published:** 2019-02-14

**Authors:** Aaro Toomela, Sven Nõmm, Tiit Kõnnussaar, Valdar Tammik

**Affiliations:** ^1^School of Natural Sciences and Health, Tallinn University, Tallinn, Estonia; ^2^Faculty of Information Technology, Tallinn University of Technology, Tallinn, Estonia

**Keywords:** behavioral indicators, skin conductance level, movement mass, hard-to-read people, person oriented approach

## Abstract

It is commonly assumed that behavior reflects the mental states of individuals. However, recent attempts to detect human states of mind via behavioral indicators have not always been successful; behavioral indicators may be unreliable and invalid. In this study we show that one of the common behavioral indicators, change in the overall amount of movement, correlated well with changes in the skin conductance level (SCL) at the group level, which reflects changes in arousal. At the individual level, however, changes in the SCL were related to movement patterns only in about half of the individuals. It is also noteworthy that the level of movement-SCL correlation was very highly predictable by certain social and cognitive characteristics of the individuals. Our results suggest that behavioral indicators may in many cases fail to predict mental states at the individual level.

## Introduction

In everyday life we often need to predict future behaviors and states of mind of other persons. Shopkeepers try to spot thieves before they steal something, airport security looks for signs to identify potential terrorists, each of us is interested in recognizing liars or noticing that loved ones are stressed. Usually people make such predictions without knowing exactly on what basis they are doing it. In certain areas, however, complex explicit procedures have been developed for identifying people with certain intentions. For instance, the United States Transportation Security Administration (TSA) runs a program called Screening Passengers by Observation Technique to identify potential terrorists. After spending close to a billion US$, the program seems to have failed. It has been concluded that the human ability to accurately identify deceptive behavior based on behavioral indicators is the same or slightly better than chance (Bond and DePaulo, 2006; Lord, 2013). TSA seems to disagree with such a conclusion (Neffenger, 2015). It is noteworthy that errors in detecting deception do not emerge because people rely on wrong cues; the problem seems to be in behavioral cues to deception that are not reliable (Hartwig and Bond, 2011).

Measures other than behavioral indicators can be used for detecting intentions and mental states, among them are physiological responses, speech analysis and measurement of brain activity. Some procedures, such as measurement of brain event-related potentials or certain forms of interviewing, may be more accurate, yet their application in real-life situations, as in airports with millions of passengers whose intentions must be estimated quickly, is not realistic because of high cost and ethical considerations (see for state-of-the-art reviews of different procedures, Granhag et al., 2015; Meijer et al., 2016). So, the first screening procedure for detecting intentions in most real-life situations still has to rely on behavioral indicators, which, as the studies referred above are showing, are not very reliable.

Recognition of intentions and other mental states concerns not only security professionals but all people. It has been found in numerous studies that both laypeople and presumed lie experts, such as police officers or business managers, endorse a faulty stereotype about the characteristics of deceptive behavior. Among other incorrect beliefs, people tend to think that deception is related to posture shifts, self-manipulations and fidgeting (Hartwig and Granhag, 2015). The authors of the latter review also suggest that such a pattern of motor activities may actually indicate stress, nervousness, discomfort, shame and guilt erroneously assumed to be related to lying. It follows that motor restlessness is a non-specific behavior that can accompany nervousness, stress or high arousal independently of the specific causes of those states. It is noteworthy that people do not rely so much on erroneous cues in detecting lies even if they hold faulty stereotypes; low detection level can be attributed to weakness of behavioral cues to deception (Hartwig and Bond, 2011).

Nevertheless, motor overactivity might be an informative sign in everyday human interaction showing that a person is distressed. Indeed, there are numerous studies showing that fidgeting, postural shifts and other signs of motor overactivity are related to electrodermal activity (EDA), which is a valid measure of autonomic nervous system activity. EDA can be used as an index of arousal, emotion and other similar states (Jurich and Jurich, 1974; Heerey and Kring, 2007; Gunther et al., 2013). Furthermore, EDA is related not only to overt motor behavior but also to activation of several brain regions, including motor regions (Fredrikson et al., 1998; MacIntosh et al., 2007; Mochizuki et al., 2009).

Fidgeting and other motor signs are relatively easy to recognize. EDA is measured by changes in the skin conductance level (SCL) caused by changes in sweating. Sweating can also be identified by observation and has already been related to emotional arousal a long time ago (Fere, 1899). Thus, not surprisingly, fidgeting, rubbing or wringing hands and sweaty palms are among the behavioral indicators in the list of suspicious signs in the TSA’s behavior checklist (Winter and Currier, 2015).

Now it may seem that if nothing else, humans could manage quite well in recognizing the arousal level or general emotional state of others by just observing their overall movement patterns, yet it is not necessarily so. The problem may be related not so much to the human ability to recognize such patterns but rather to individual differences in expressing motor restlessness or any other sign of discomfort, deception or whatever other mental state we would be interested in detecting. It has been found, for instance, that lies are harder to detect when a liar has had time to prepare for lying; it is easier to detect lies when the receivers have had previous exposure to senders (Bond and DePaulo, 2006). Next, some people suppress either emotional response or expression of their emotions more than the others; such suppression can be spontaneous rather than effortful (Gross and John, 2003; Egloff et al., 2006; Gyurak et al., 2011; Chen et al., 2017) and culturally learned rather than innate (Murata et al., 2013). Thus, suppressors may have learned to do so even not realizing they are suppressing expression of their emotions. Such individual differences in expressing mental states are in agreement with findings suggesting that when telling lies and truths, some people are significantly more detectable than others (Bond and DePaulo, 2008).

Altogether, available evidence points to a possibility that behavioral indicators often used as signs of certain mental states might be either missing or misleading in a subset of people. This hypothesis is further supported by studies showing that even the most advanced lie-detection technologies are not sufficiently accurate in detecting deception at an individual level (Mameli et al., 2017).

In this experiment we explore how changes in arousal level are expressed in overall motor activity. On one hand, we expect changes in arousal level to be related to changes in motor activity while on the other hand, as the results of abovementioned studies on individual differences in expressiveness of mental states suggest, we expect to find that changes in arousal level are going to be expressed in motor activity patterns in only a subset of individuals. There should be some easy-to-read persons whose arousal level is reflected in their motor activity and hard-to-read persons in whom the arousal-activity relationship does not appear.

The relationship in arousal level and motor activity has been found in many group level studies. If our results would support the idea that changes in arousal level are expressed in changes in motor activity only in a subset of individuals, we would need to demonstrate that the individual differences we find are real and not caused by some technical problem of the study instead. We assume that if individuals do not express their arousal state in motor activity then they might be hard-to-read also in other aspects of mental states. If this is the case, then there must be psychological characteristics that distinguish hard-to-read people from easy-to-read people. We found some such characteristics in the literature. First, there is evidence that suppressing emotional expressions is related to interpersonal functioning (Butler et al., 2003; Gross and John, 2003; Peters et al., 2014). It has also been found that negative personality characteristics are attributed to suppressors (Tackman and Srivastava, 2016). Correspondingly, we measured different aspects of interpersonal functioning. Next, it has been found that physical attractiveness is related to social status (Mailend, 2010; Gordon et al., 2013). We hypothesized that people with lower social status tend to suppress expression of their mental states, so, we also measured physical attractiveness of the participants. Finally, gender (Stewart and McDermott, 2004) and cognitive abilities (Weinberger, 2014) are related to social skills and relations. We hypothesized that if we have correctly distinguished easy-to-read individuals from hard-to-read individuals, then in our experiment at least some of these factors should be related to expressiveness of the arousal level in motor activity.

## Materials and Methods

### Measures and Tasks

#### Movement and Arousal Measures

We were interested in how well changes in motor activity could inform us about the changes in arousal level measured by changes in SCL. There are different ways to objectively describe motor activity recorded by a motion sensor. We have developed several novel measures, among them is trajectory mass (TM), that has intuitively clear meaning and characterizes the state of movement very well. Essentially, TM describes how much a certain joint has moved in a time unit either in relation to another joint or to the previous position in space.

Formally the TM is calculated as follows: Denote *J* = {*j*_1_*, j*_2_*,..., j*_n_} be the set of the joints of interest. Let *T*_ji_ be the length of the trajectory of the joint *j*_i_ observed during the motion. Define TM as the sum of the trajectory lengths computed of each joint of the set *J*:

$$T_{j}=\sum_{i=1}^{n}T_{\mathrm{ji}}$$

In other words, TM is a sum of traveled distances summarized over all joints of interest in a time unit. We have shown that such measures distinguish highly reliably skilled movements from unlearned movements (Nõmm and Toomela, 2013; Nõmm et al., 2013) and movement patterns of patients with a Parkinson’s disease from those of healthy persons (Nõmm et al., 2016). In this study we divided the entire time of the experiment into 86 time segments of equal length of about 13 s; the position of joints was recorded 300 times during this 13 s time segment and respectively, 299 changes were summed for each time segment. The traveled distance of a joint was measured between each two adjacent recording points. The summary measure for one time segment reflects the sum of all the distances traveled during the time segment. The decision to define a time segment with 300 recording points was to some degree arbitrary. In shorter periods, too much noise would contribute to the variability of the measure and in longer periods real-time meaningful variability of the measure would disappear. Altogether we got 86 data points (one for each time segment) for each participant. In this study we calculated average TM for dominant hand (TMDH, there were three joints taken into account: wrist, elbow and shoulder). The higher the TMDH score, the more the dominant hand had moved during 13 s.

Unlike the measured value of SCL, which is recorded for each instance of time, parameters of the TM are associated with time intervals. In order to compare SCL to the TM we derived the parameters describing the amount of the SCL changes for a given time interval in a similar way to TM: Denote *C*_i_ amount of changes of SC during a time interval *i*. In other words, TM for SCL is a change of the SCL during two adjacent recording points. We calculated a summary of changes of the SCL between adjacent recording points (TMSCL) during the same 13 s time frames as the TMDH. Variation in the TMSCL reflect changes in arousal level during the study session. The higher the TMSCL score, the more SCL changed during 13 s.

#### Social Status Measures

We assumed that individuals with high and low expressiveness of their arousal level can be distinguished in our study. We were not aware of any studies where such a result would have been reported. Nevertheless, there have been studies of people who tend to suppress the expression of their emotional state. These studies have shown that one area where suppressors can be distinguished from non-suppressors is interpersonal functioning (see Introduction, above). So, we selected measures of different aspects of interpersonal functioning to study further individual differences in arousal expressiveness.

We used several measures that allowed us to estimate the quality of interpersonal functioning of the participant. First, *popularity* of all participants was measured. All pupils of the class were asked to name three classmates they would definitely invite to their birthday and invite to go hiking together. They were also asked to name three classmates they definitely would not invite to birthday or hiking. Both positive and negative nominations were summed for each participant. As the number of pupils in classes varied considerably, we adjusted the nomination counts for the class sizes. Thus, popularity of a participant was characterized by *classmate positive popularity index* and by *classmate negative popularity index*.

Second, we estimated whether the participants are characterized by constructive or deconstructive group initiatives. To estimate the *constructive group initiative* aspect of interpersonal functioning, we asked the pupils of the class to nominate three classmates who would manage best with organizing a ceremony to celebrate the school anniversary, to nominate two classmates who would manage best to organize the next class excursion and to nominate two classmates who would invite the class not to leave the school and skip the last course. Each pupil in the class was characterized by a sum of positive nominations; as chances to be nominated were bigger in smaller classes, the sum of nominations was adjusted for the class size. The *deconstructive group initiative* was measured similarly by asking to nominate two classmates who would invite the class to leave the school and skip the last course. Thus, constructive and deconstructive group initiative of a participant was characterized by *classmate constructive group initiative index* and by *classmate deconstructive group initiative index*.

What to consider as constructive or deconstructive group initiatives may be perceived differently by peers and by adults. We asked three subject teachers who were familiar with the class to characterize all pupils and to mark each pupil on whether he or she would initiate positive/constructive activities in the class or negative/deconstructive activities in the class. Pupils with low initiative levels were not nominated. We summed the positive nominations and the negative nominations separately. Thus, teacher’s perception of group initiative levels was characterized by *teacher constructive group initiative nominations* and *teacher deconstructive group initiative nominations*. In earlier studies the social status measures we use in this study have been found to be reliably related to academic success and several interpersonal characteristics of pupils (Mailend, 2010; Tago and Ots, 2010; Reek, 2012).

Finally, it is known that physical attractiveness is one of the factors that determines the quality of social relationships (Mailend, 2010; Gordon et al., 2013). We made group photos of all classes and asked four adults to indicate the most beautiful and the least beautiful pupil in each of the classes (we made absolutely sure that the referees opinions remained anonymous). We summed the nominations into *very attractive nominations* and *very unattractive nominations*, respectively. In earlier studies this measure was found to be reliable related to academic success and several interpersonal characteristics of pupils (Mailend, 2010).

#### Cognitive Measures and Self-Esteem

Suppression of the expression of mental state requires recognition of those states as well as control of behavior. We expected that individuals with higher level of cognitive abilities may be better at controlling their expressiveness. Cognitive abilities of the participants were measured with two tests. General cognitive (also called non-verbal reasoning) ability was measured by the D and E-sets of the *Raven’s Standard Progressive Matrices* (Raven, 1981; Lynn et al., 2003). A participant was shown one by one 24 matrixes with a missing part and was asked to select one of the eight response options to complete the matrix.

For measuring verbal reasoning skills, the *Word Guessing Test* (Männamaa et al., 2008) was used. We used an eight-item version of the test where the respondent is asked to infer the name of the verbal concepts on the basis of the clues (e.g., “planet” was a correct response to the test item “What is a celestial body that does not produce light and revolves around the Sun?”). Performance on this task requires not only vocabulary but also integration of fragments of knowledge and logical deductions.

Finally, considering that suppressors are usually attributed negative personality characteristics (Tackman and Srivastava, 2016), we assumed that suppressors may be characterized by lower level of self-esteem. Participants’ *general self-esteem* was measured by the Estonian version (Pullmann and Allik, 2000) of the ten item Rosenberg Self-Esteem Scale (Rosenberg, 1965). Items were coded on a 3-point Likert scale from 1 (*strongly disagree*) to 3 (*strongly agree*). The ratings for the statements were summed, with negative statements reverse coded. Higher scores represented higher levels of the self-reported general self-esteem.

### Participants

Sixty ninth-grade adolescents (28 males, 32 females) were recruited from 13 schools across the country. Schools differed in location (town, rural), size and level of the graduates according to the National Examination results. Participants ranged in age from 13 to 15 years, with an average age of 14.05 years (*SD* = 0.29). This sample comprised a subsample of a large scale study of child development at primary school. Participants for this study were selected to represent as diverse as possible level of academic achievement and demographic backgrounds. Sample size was determined before any data analysis.

### Procedure

The participants had been studied with a battery of psychological tests and questionnaires before the experiment reported in this paper (see Kikas and Toomela, 2015, for the description of the project). In this experiment, the participants were studied individually. They were asked to play a *computer-game* (written in Python language; designed for study by the third author) and respond on different stimuli with mouse clicks in a 19 minute session. The session contained 13 segments that were composed intermittently to either increase the stress level (such as programmed overspeeding of a game or a frightening video clip) or to relax (nature views). The segments of the game were selected on the basis of a pilot study where participants (*N* = 10; participants with different age and education levels) reported their experiences about the computer game and choices for segments. A high level of stress or emotional state was reported about the game and expectedly stressful segments. A low level of stress was reported about the nature view segment. The participants were not informed about the aim of the study to observe relationships between motor activity and arousal level. This information was provided after the study.

During the session two objective measures were recorded. First, motor activity was continuously recorded with the markerless and easy to use Microsoft Kinect motion sensor, connected to a PC. Kinect provides 3D coordinates of an artificial skeleton with 19 body joints and a head, with sampling time of 30 Hz. Control of the Kinect sensor, data acquisition and storage were performed by means of a specially developed application. This application allows us to read the data from the sensor, marking the beginning and the ending times for each motion of interest and storing the data for further analysis. MATLAB was used to analyze the data. As participants were not moving around but sitting by a table, all their movements could not be recorded. We limited our data to the movements of the dominant hand of the individual.

Second, to describe EDA, changes in SCL were recorded with BIOPAC Systems Inc. MP150 data acquisition and analysis system with GSR100 amplifier and Acq*Knowledge* software. Disposable isotonic snap electrodes EL507 were used; the electrodes were attached to the hypothenar eminence of the non-dominant hand. The results were recorded at 200 Hz frequency during the whole session. Low pass filter was applied to smoothen the data and eliminate non-specific electrodermal reactions. After recording, the Kinect and EDA data were synchronized at 22.6 Hz.

## Results and Discussion

We were interested in how well changes in motor activity could inform us about the arousal level measured by changes in SCL. We calculated trajectory masses (TM) of the relevant parameters. In this study we calculated average TM for dominant hand (TMDH) and average TM for the SCL time curves (TMSCL) during the same time frames.

### Group-Level Relationship Between Motor Activity and Arousal Level

If our measures are valid and reliable, the TMDH should be significantly correlated with the TMSCL over the entire session. We calculated TMDH and TMSCL scores for the entire experiment. The correlation between the variables was substantial: *r* = 0.56; *p* < 0.0001; 95% CI [0.35, 0.71]. Thus, we have replicated the results of numerous other studies where changes in motor activity have been found to be related to changes in arousal level. Yet, as we are going to show next, this conclusion would be, in an important sense, incorrect.

### Individual-Level Relationships Between Motor Activity and Arousal Level

In many sciences today, it is common to assume that it is possible in statistical data analysis to generalize interindividual variation between cases to the understanding of intraindividual variation within single cases. This assumption, however, is wrong for mathematical and methodological reasons (Molenaar, 2004; Toomela, 2010). So, we decided to analyze data at the individual level as well. We divided the entire session into 86 time-units and calculated TMDH and TMSCL scores for each unit for each participant. Then we calculated correlations between TMDH and TMSCL, that is motor-arousal correlations (MAC), for each participant separately. It turned out that at the individual level of analysis the results are very different. Intraindividual correlations varied between -0.023, 95% CI [-0.23, 0.19] to 0.694, 95% CI [0.57, 0.79] (average correlation 0.267); in 5 participants out of 60 the correlation was equal or higher than 0.50, 95% CI [0.32, 0.64] and in 25 cases equal or higher than 0.30, 95% CI [0.1, 0.48]. Thus, on the one hand, we found that in many cases motor activity is not substantially related to arousal level. On the other hand, in a subgroup of studied individuals the observed effect sizes of the correlations are at least moderate. So, we can conclude that there are also people in whom motor activity is reliably associated with the arousal level. We can also understand why motor activity level intuitively seems to be a reliable indicator of a person’s emotional or arousal state. Our data show that there are people whose arousal level varies together with changes in their motor activity patterns. Such people can give us positive evidence for the assumed relationship. And there are other people whose motor activity is not informative. In such cases we just get no idea what the actual state of a person might be and therefore we do not have evidence against our impressions. Selective ignoring of the evidence against the universal motor activity-mental state relationship would be supported by so-called confirmation bias, the human tendency for inappropriate bolstering of hypotheses or beliefs of whose truth is in question (Nickerson, 1998; Talluri et al., 2018).

### Reliability of Our Findings

The data we have provided so far are not sufficient to support the conclusions we made. We must be certain that, in cases of statistically insignificant correlations at the individual level, the small size of the correlations was not caused by equipment failure or some other methodological problem.

First, it is possible that the methods we used did not cause changes in arousal levels in all participants as we expected. That could explain very low MACs we found in many individuals: It is possible that MAC was high only in cases when arousal level changed considerably, and MAC was low if the arousal level did not change much. In that case MAC should be positively correlated with the summary TMSCL over all the session. The correlation, however, was very low (*r* = -0.087; *p* > 0.5; 95% CI [-0.33, 0.17]).

### Validity of Motor-Arousal-Correlation Measure

Our conclusions can be valid only if MAC is a valid and meaningful dimension of individual differences. To ground our conclusions further, we tested the validity of MAC in another way. If MAC is a valid measure, it should correlate with other relevant attributes of individuals. It has been found that people who suppress emotional expressions have also difficulties in interpersonal functioning (Butler et al., 2003; Gross and John, 2003; Peters et al., 2014), this might be related to negative personality characteristics of suppressors attributed to them by others (Tackman and Srivastava, 2016). Thus, measures of social relationships should be related to MAC. Following the same line of evidence, physical attractiveness also contributes to the quality of social status and social relationships already in adolescents. Good-looking adolescents have social and academic advantages whereas unattractive teenagers are often socially stigmatized, they may suffer from social isolation, low self-esteem and depression (Gordon et al., 2013). Finally, both gender (Stewart and McDermott, 2004) and cognitive abilities (Weinberger, 2014) are related to social skills and relations.

We collected data about cognitive abilities (non-verbal and verbal abilities), general self-esteem and social relations (popularity, group initiative and physical attractiveness). We measured separately positive (most popular, constructive group initiative, most attractive) and negative (most rejected, deconstructive group initiative, the least attractive) aspects of interpersonal functioning. The pattern of pairwise correlations between MAC and other measures (see Table 1 for details) both supported the idea that MAC is a valid dimension of individual differences and gave more understanding of what kind of persons tend to be easy to read.

**Table 1:** Correlations between MAC, interpersonal, and psychological measures (*N* = 60).

Interpersonal and psychological measures	MAC		
	
	Correlation	*p*-value	95% CI
Classmate positive popularity index	0.294	0.022	0.04, 0.51
Classmate negative popularity index	-0.070	0.597	-0.32, 0.19
Classmate constructive group initiative index	0.395	0.002	0.16, 0.59
Classmate deconstructive group initiative index	-0.036	0.784	-0.28, 0.22
Teacher constructive group initiative nominations	0.448	0.000	0.22, 0.63
Teacher deconstructive group initiative nominations	-0.251	0.053	-0.47, 0.00
Very attractive nominations	0.309	0.016	0.06, 0.52
Very non-attractive nominations	-0.089	0.498	-0.34, 0.17
Gender	0.257*	0.004	
Word Guessing test	-0.258	0.046	-0.48, -0.01
Raven’s standard progressive matrices	0.030	0.822	-0.23, 0.28
Rosenberg self-esteem scale	-0.253	0.051	-0.48, 0.00


*^∗^Kendall Tau-b.*

As to the measures of social relations, a clear pattern emerged: positive-constructive aspects of social relations were positively correlated with MAC: classmate positive popularity index, classmate constructive group initiative index, teacher constructive group initiative nominations, and very attractive nominations. At the same time, from negative-deconstructive characteristics, only one teacher deconstructive group initiative nominations was low but close enough to reach the acceptable level of significance; in all other cases the correlations were also negative but very low.

Gender (male = 0; female = 1) was positively correlated with MAC showing that women tend to be easier to read. From cognitive abilities, we found low negative correlation between verbal abilities and MAC, whereas correlation with non-verbal ability measure was close to zero. Finally, the correlation between general self-esteem and MAC was also low and close to reach the acceptable level of significance.

### Psychological Portrait of Easy-to-Read People

Correlations between MAC and other measures allow to propose a tentative psychological portrait of easy-(or hard)-to-read people (obviously in terms of motor-arousal relationship only). Before doing that, however, we have to consider the fact that the measures we used characterize partly overlapping qualities. We conducted a forward-stepwise (*F* to enter = 2.00) multiple regression analysis to overcome that problem. The results of the analysis are provided in Table 2.

**Table 2:** Summary of the forward stepwise multiple regression analysis for variables predicting motor-arousal correlation level (*N* = 60).

Variables in the model	β	*t* (53)	*p*
Teacher constructive group initiative nominations	0.269	2.37	0.021
Rosenberg self-esteem scale	-0.326	-3.26	0.002
Very attractive nominations	0.293	2.95	0.005
Classmate constructive group initiative index	0.309	2.81	0.007
Word Guessing Test	-0.264	-2.31	0.025
Raven’s Matrices	0.169	1.48	n.s.


The analysis revealed that 49% of the MAC variability can be statistically explained by a few independent variables [*R*^2^ = 0.488; *F*(6,53) = 8.43; *p* < 0.00001]. Our data suggest, as can be seen in Table 2, that easy-to-read persons express constructive initiative, are physically more attractive but with lower self-esteem and with lower verbal abilities. General cognitive ability (Raven’s Standard Progressive Matrices) also entered the model but did not contribute significantly to prediction of the dependent variable.

### Limitations of the Study

This study has some limitations. As to the positive side, we studied a sample of adolescents with very wide variability in socio-economic background and cognitive and personality characteristics. Such high variability is harder to achieve with adult samples. However, we cannot be certain that movement-arousal correlations observed in this study would also apply to adults. Several studies have demonstrated that SCL is a reliable and valid measure of mental states in children (e.g., Schupak et al., 2016; Najafpour et al., 2017) and even in infants (Ham and Tronick, 2008). But we are not aware of any study where intraindividual SCL-movement pattern relations had been studied longitudinally from adolescence to adulthood. Further studies are needed to explore this issue.

Another question is related to using the movement data about the dominant hand. It has been found that SCL is to some degree lateralized and the lateralization varies also depending on the lateralized hemispheric activation (Lacroix and Comper, 1979; Roman et al., 1992; Bracco et al., 2017). We could not place the movement sensor so that movements of both hands could be observed at the same time. We also could not attach the SCL sensor to the dominant hand that was used for manipulating the mouse. So, we cannot rule out the possibility that the observed SCL-movement relations could be different if both SCL and TM would have been measured bilaterally. Again, further studies are needed to explore this issue.

## Conclusion

Altogether, we can conclude that there might be many individuals among us whose motor activity pattern does not reflect their arousal level. Further, there is no evidence to suggest that this conclusion can be made only about motor activity-arousal level relations. There can be a large number of people whose behavior does not reflect their mental state. It is also interesting that in our study, easy-to-read people, as correlations of the MAC with measures of social relations revealed, tended to have good intentions and those with bad intentions are often hard-to-read. Belief that behavioral indicators are useful may be based on two complementary sources of misleading information. On the one hand, most scientific research in this area is conducted only at the group level; our data show clearly that group-level generalization may fail at the individual level of analysis. On the other hand, our belief in such indicators may be based on everyday observations of individuals whose behavior actually does reflect their mental state; we are just not able to realize that there are numerous people around us who are hard to read.

## Ethics Statement

An ethics approval was not required for this study as per institutional guidelines and Estonian regulations. Passive parental consent was obtained in all cases after all necessary information about the study was provided to the parents.

## Author Contributions

AT, SN, TK, and VT contributed conception and design of the study. TK and VT collected the data and organized the database. AT wrote the first draft of the manuscript. All authors contributed to manuscript revision, read and approved the submitted version.

## Conflict of Interest Statement

The authors declare that the research was conducted in the absence of any commercial or financial relationships that could be construed as a potential conflict of interest.
